# Successful Radical Pneumonectomy for a Primitive Neuroendodermal Tumor in the Lung: A Case Report and Review of the Literature

**DOI:** 10.3389/fsurg.2021.667467

**Published:** 2021-04-28

**Authors:** Bingqing Yue, Peng Chen, Pan Yin, Jiankai Wang, Fanying Liu, Duo Zhao, Jingyu Chen, Hua Jiang

**Affiliations:** ^1^Wuxi Lung Transplant Center, Department of Thoracic Surgery, Wuxi People's Hospital Affiliated to Nanjing Medical University, Wuxi, China; ^2^School of Medicine, Henan University of Traditional Chinese Medicine, Zhengzhou, China; ^3^Department of Thoracic Surgery, Yanggu People's Hospital, Liaocheng, China; ^4^Department of Thoracic Surgery, Shandong Provincial Hospital Affiliated to Shandong First Medical University, Jinan, China; ^5^Department of Surgery, The First Affiliated Hospital of Henan University of Traditional Chinese Medicine, Zhengzhou, China

**Keywords:** primitive neuroendodermal tumors, pneumonectomy, prognosis, diagnosis, small round cell malignant tumors

## Abstract

Peripheral primitive neuroendodermal tumors (PNETs) and Ewing's sarcoma belong to the Ewing family of tumors and are small round-cell malignancies originating from spinal cord cells. These tumors account for 5% of all small round-cell malignant neoplasms. PNETs that arise from the lung parenchyma without pleural or chest wall involvement are very rare. We report a case of an adult female with a large pulmonary PNET who had given birth just 1 month prior to the diagnosis. She had cough and expectoration for 6 months, and the preoperative examination showed no metastases. Thus, we performed radical pneumonectomy and lymph node dissection. The patient recovered well without surgical complications and was discharged 7 days after the surgery. Postoperative pathology confirmed that the tumor was a small round-cell malignancy, and the tumor cells were positive for CD99, Friend leukemia virus integration 1 (FLI-1), and neuron-specific enolase (NSE), which was consistent with the diagnosis of a PNET. For primary large pulmonary PNETs, radical pneumonectomy may be a safe surgical method, worthy of further application in clinical practice.

## Introduction

Primitive neuroendodermal tumors (PNETs) are small round-cell malignancies originating from the neural crest. PNETs can occur at any age but are most common among adolescents and adults under the age of 35 (1). The male-to-female ratio is ~1.5:1 (2). The overall incidence of PNETs is low, and the prognosis is poor. PNETs arise more frequently in the bone and soft tissue and are very rare in other parts of the body. However, there have been reports of PNETs in the kidney, liver, urinary bladder, myocardium, adrenal glands, pancreas, and female genital tract (3–9). In 1979, Askin et al. (10) first reported a PNET that occurred in the chest, which is currently referred to as Askin's tumor. However, cases of pulmonary PNETs without chest wall or pleural involvement are extremely rare worldwide. Herein, we report a case of a large pulmonary PNET in an adult female who gave birth just 1 month prior to the diagnosis.

## Case Presentation

A 30-year-old female patient presented with a 6-month history of cough and expectoration, during which time she felt palpitations and sometimes, chest suffocation. She denied fever, dyspnea, asitia, cyanosis, hoarseness, hemoptysis, or night sweats. She had a baby just 1 month prior to the examination and had pain in her right rib 7 days prior. She denied a history of smoking and drinking. Because of ectopic pregnancy, she underwent surgery 7 years ago, during which she lost blood and received transfusion therapy.

Physical examination revealed that the breath sounds in the left lung field were weakened. Laboratory studies showed that her white blood cell count was 12.4 × 10^9^/L; the hemoglobin level was 97 g/L; and the platelet count was 555 × 10^9^/L. D-dimer level was 8.37 mg/L. Tumor marker analysis showed that the squamous cell carcinoma antigen had increased to 3.4 ng/mL, and her CYFRA211 level had increased to 90.2 ng/mL. Pulmonary function test showed maximum voluntary ventilation (MVV) was 67.98 L, accounting for 63.1% of the predicted value; forced expiratory volume in 1 s (FEV1) 2.09 L, accounting for 72.3% of the predicted value. Contrast-enhanced computed tomography (CT) scans revealed a large soft tissue mass in the lower left lobe of the lung; the maximum cross-section was ~11.7 × 15.4 cm. The density of the mass was uneven and showed inner crumb calcifications. The solid parts of the mass were enhanced with moderate heterogeneity. The bronchus of the lower lobe of the left lung was truncated. The bilateral axillary lymph nodes were enlarged, and multiple enlarged lymph nodes were observed in the left hilus of the lung. Pleural effusion was observed on the left side of the chest cavity. CT results are considered as left lower lobe tumor with mediastinal and left hilar lymphadenopathy. Chest radiography showed a patchy shadow in the lower lobe of the left lung. The left hilus of the lung was expanded, and there was a left pleural effusion.

To identify the location and the type of the tumor, a bronchoscopic examination was performed. Bronchoscopy showed that the basal segment of the lower lobe of the left lung was entirely blocked by a tumor, which had a cauliflower-like appearance. The remaining bronchial mucosa of the lower lobe of the left lung was slightly congested, and the ridge was widened. Brush detection revealed a few cells with large bodies, arranged in groups, which is may be considered as lung cancer. A bone scan and abdominal ultrasonography showed no metastases, and the cardiopulmonary function of the patient was adequate; therefore, we performed surgery. The patient provided informed consent and signed the consent form.

**Figure 1:**
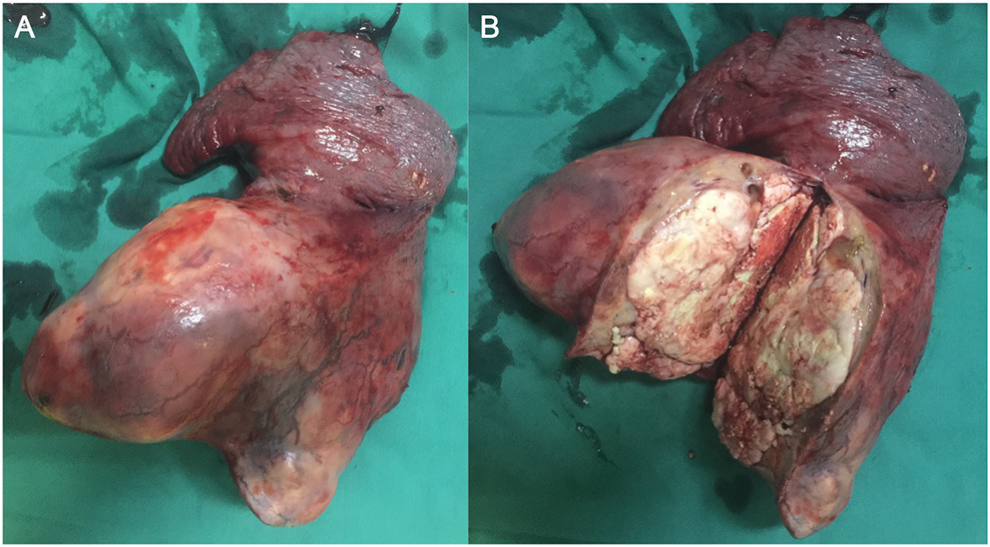
Intraoperative images. **(A)** Excised left lung. **(B)** The tumor.

During surgery, the left lower lobe of the lung was found to have adhered to the thoracic cavity, and a hard mass involving the entire left lower lobe and inferior pulmonary veins was present, without metastatic nodules in the pleura. To excise the upper and lower pulmonary veins, we opened the pericardium and successfully resected the total left lung and cleaned the 4, 5, 7, 9, and 10th nodes (Figure 1). We reinforced the bronchial stump with 4-0 non-traumatic absorbable thread by continuous back and forth sutures. The bleeding volume was 500 mL, with a 400-mL blood transfusion. The operation lasted for ~4 h. Because the patient was young and not at risk of cardiovascular and cerebrovascular diseases before the operation, she was directly transferred to the general ward after operation.

**Figure 2:**
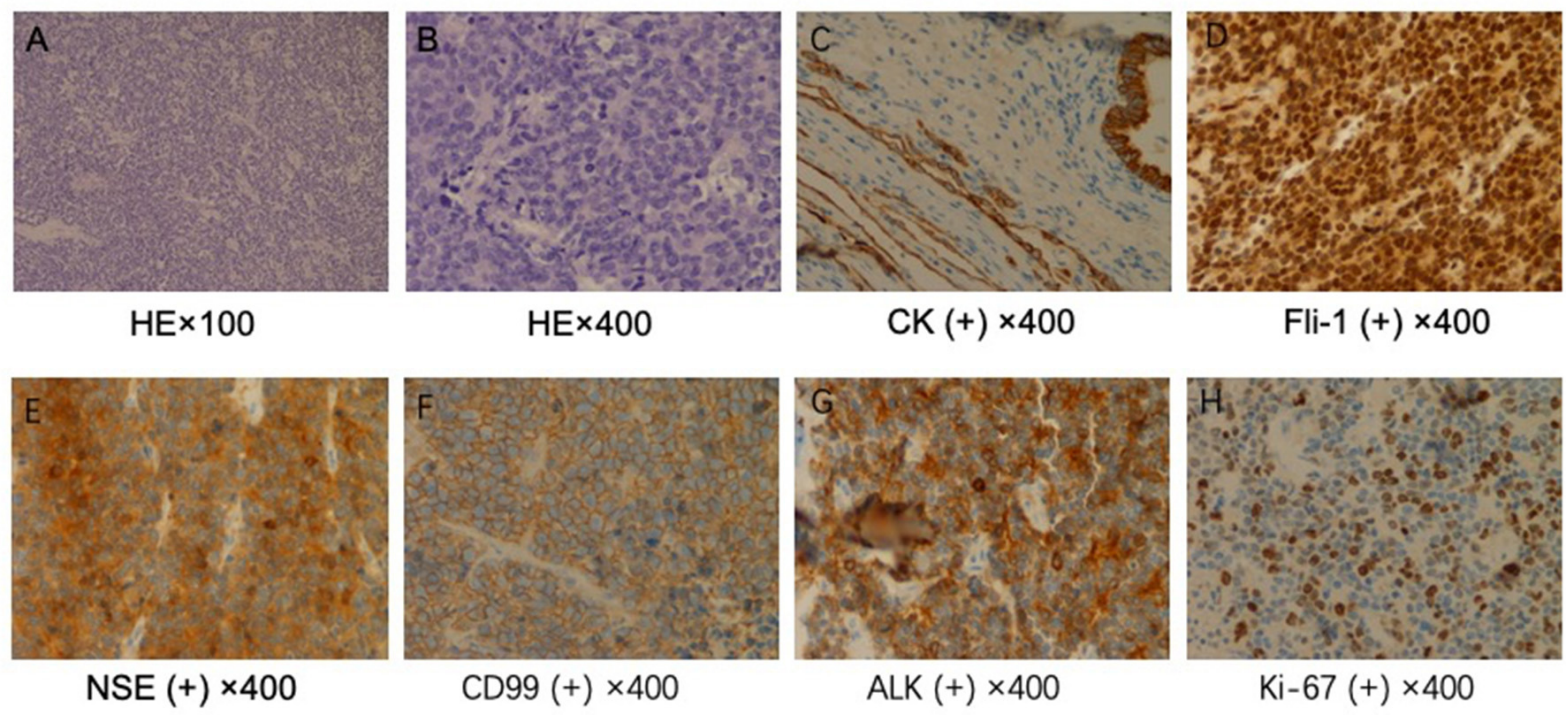
Pathological examination. **(A)** ×100 and **(B)** ×400. Hematoxylin and eosin staining shows a large number of small, round malignant cells. Immunohistochemical staining for **(C)** creatine kinase (CK), **(D)** Friend leukemia virus integration 1 (FLI-1), **(E)** neuron-specific enolase (NSE), **(F)** CD99, **(G)** anaplastic lymphoma kinase (ALK), and **(H)** KI-67.

The patient received preventive antibiotic and supportive treatments after the operation. The chest tube was removed on the fourth day after the operation. Postoperative pathology confirmed that the tumor was a small round-cell malignancy with a cross-section of 12 × 11 cm. The tumor was adjacent to the visceral pleura, and a bronchial tangent and nodes were not found in the tumor. One of the lymph nodes showed extensive necrosis. Immunohistochemistry revealed that the tumor cells were positive for creatine kinase (CK), Friend leukemia virus integration 1 (FLI-1), neuron-specific enolase (NSE), CD99, and anaplastic lymphoma kinase (ALK). The cell proliferative index (KI-67) was ~50%. The tumor cells were negative for cytokeratin 7, P40, thyroid transcription factor-1, CD56, chromogranin A, vimentin, CD30, CD43, α-inhibin, myeloperoxidase, S-100, and CD138 (Figure 2). The patient recovered well and was discharged 7 days after surgery

## Discussion

Peripheral PNETs and Ewing's sarcoma belong to the Ewing family of tumors and are small round-cell malignancies originating from spinal cord cells and accounting for 5% of all small round-cell malignant neoplasms (11). Until now, only few pulmonary PNETs have been reported in the literature (12–14). Common clinical manifestations include chest pain, cough, and fever. CT scans often detect large lumps. Our case was mainly characterized by cough and chest pain; the tumor diameter, as shown by CT, was ~12 cm.

Pulmonary PNETs have no specific symptoms or imaging manifestations, and the diagnosis mainly relies on pathological and immunohistochemical findings or genetic techniques. Pathologically, PNETs are composed of differentiated or poorly differentiated small, blue, round cells that are diffuse, leaf-shaped, or nested and separated by fibrous vessels. Necrosis, hemorrhage, and cystic changes often occur. Some tumor tissues can be observed in the Homer–Wright or Flexner–Weinsteiner structure. PNETs are often strongly positive for CD99, which is a cell-surface glycoprotein encoded by the heterochromatin *CD99* gene (also known as *MIC2*) (15). In addition, studies have found that NSE, CD56, and vimentin can be present at different levels (16) and that the FLI-1 protein may also be of great value in diagnosing PNETs (17). At least two of these markers should be positive to diagnose a tumor as a PNET (16). Genetic diagnosis involves the detection of the *t*(11;22)(q24;q12) translocation, which is present in 90–95% of PNET cases (18). Our case was positive for CD99, FLI-1, and NSE. No tumor was discovered elsewhere, and therefore, the diagnosis was a pulmonary PNET.

Current treatments include surgery, chemotherapy, and radiation therapy; however, PNETs are still considered highly aggressive malignant tumors and metastasize rapidly, with a poor prognosis. The 5-year survival rate is <25% (19). Moreover, the efficacies of targeted therapies for PNETs remain uncertain.

Currently, the most commonly used chemotherapy drugs to treat PNETs include cyclophosphamide, vincristine, doxorubicin, etoposide, and ifosfamide (20). Demir et al. (21) reported that the 5-year survival rates of patients who underwent radical surgical resection or palliative surgery were 56 and 25%, respectively (*P* = 0.13). Five-year survival of patients treated with neoadjuvant chemotherapy was 77%, compared to 37% in the untreated group (*P* = 0.22). Chemotherapy can improve the rate of survival after radical surgery (21). A recent study has demonstrated that angiogenesis can promote the growth and metastasis of solid tumors (22). Li et al. (23) reported a patient who was treated with thalidomide because of the poor effect of conventional therapy and who was in a good condition, without disease progression for 15 months. To date, no successful pneumonectomy of such a large pulmonary PNET has been reported. Our patient underwent radical tumor excision and lymph node dissection. However, because of financial problems, she did not receive follow-up chemoradiotherapy and was discharged 7 days after the surgery in good condition. For large pulmonary PNETs, pneumonectomy may be a safe surgical method, worthy of further application in clinical practice.

## Data Availability Statement

The original contributions presented in the study are included in the article/supplementary material, further inquiries can be directed to the corresponding author/s.

## Ethics Statement

The studies involving human participants were reviewed and approved by the institutional ethics committee of Shandong Provincial Hospital affiliated to Shandong First Medical University. The patients/participants provided their written informed consent to participate in this study.

## Author Contributions

PC and BY designed and performed the study and wrote the paper. PY, JW, and FL collected and analyzed the data. DZ and HJ supervised the clinical research. HJ, BY, and JC revised the manuscript. All authors approved the final manuscript.

## Conflict of Interest

The authors declare that the research was conducted in the absence of any commercial or financial relationships that could be construed as a potential conflict of interest.
